# Site of Alcohol First-Pass Metabolism Among Women

**DOI:** 10.1001/jamanetworkopen.2022.3711

**Published:** 2022-03-22

**Authors:** Neda Seyedsadjadi, M. Belen Acevedo, Raul Alfaro, Vijay A. Ramchandani, Martin H. Plawecki, Blair Rowitz, Marta Yanina Pepino

**Affiliations:** 1 Department of Food Science and Human Nutrition, University of Illinois at Urbana Champaign; 2 Human Psychopharmacology Laboratory, Division of Intramural Clinical and Biological Research, National Institute on Alcohol Abuse and Alcoholism, Bethesda, Maryland; 3 Department of Psychiatry, Indiana University School of Medicine, Indianapolis; 4 Division of Nutritional Sciences, University of Illinois at Urbana-Champaign; 5 Carle Illinois School of Medicine, University of Illinois at Urbana-Champaign; 6 Carle Foundation Hospital, Urbana, Illinois

## Abstract

This cross-sectional study compares alcohol pharmacokinetics in patients who underwent sleeve gastrectomy with control participants who did not undergo surgery.

## Introduction

There is broad agreement that a fraction of ingested alcohol is metabolized before reaching the bloodstream; this is known as first-pass metabolism (FPM).^1^ The virtual elimination of FPM, seen following gastrectomy^1^ and gastric bypass,^2^ increases blood alcohol concentrations (BACs), the bioavailability of alcohol, and the risk of alcohol-related diseases.^3,4^ However, whether alcohol FPM primarily occurs in the stomach or the liver^5^ remains uncertain. While gastrectomy eliminates alcohol FPM,^1^ it is unclear whether this is because of the removal of the gastric source of FPM or the saturation of the hepatic source of FPM by the delivery of alcohol as a bolus in the absence of the gastric pylorus.^5^

Because sleeve gastrectomy (SG) reduces approximately 80% of the stomach but preserves the pylorus, we compared alcohol pharmacokinetics in patients who underwent SG with control participants who did not undergo surgery and achieved comparable time-to-peak BAC (Tmax) after drinking. This design allowed differentiation of the effects of gastric emptying rate on hepatic vs gastric FPM. In addition, to control for association of SG with potential changes in systemic alcohol elimination rates (AER) that could confound differences in the bioavailability of ingested alcohol between groups, we used an intravenous (IV) alcohol clamp that estimates AER independent of variations in alcohol absorption.^6^

## Methods

This cross-sectional study included 12 women who had SG surgery within the past 5 years at Carle Foundation Hospital (CFH) in Urbana, Illinois, and 9 women who did not undergo surgery and had equivalent age, body max index, and alcohol drinking patterns (Table and eFigure in the Supplement). Participants provided written informed consent, and National Institute on Alcohol Abuse and Alcoholism guidelines on Administering Alcohol in Human Studies were followed. The study was approved by the University of Illinois at Urbana-Champaign (UIUC) institutional review board. This study followed the Strengthening the Reporting of Observational Studies in Epidemiology (STROBE) guideline.

**Table.** Characteristics of Study Participants and Alcohol Related Variables

Characteristic	Groups			Tmax-matched groups^a^		
	Control (n = 9)	Sleeve gastrectomy (n = 12)^b^	*P* value	Control (n = 7)	Sleeve gastrectomy (n = 7)	*P* value
Clinical values						
Age, mean (SD), y	39.6 (8.5)	43.0 (7.8)	.35	40.7 (8.9)	43.9 (9.7)	.54
Weight, mean (SD), kg	91.5 (18.2)	88.6 (12.6)	.67	97.6 (15.8)	90.3 (15.0)	.39
BMI, mean (SD)	33.4 (5.4)	34.2 (4.9)	.74	35.4 (4.1)	35.3 (4.4)	.97
Fat free mass, kg	48.2 (8.4)	48.7 (4.7)	.87	50.0 (8.8)	47.9 (5.9)	.61
Time from surgery, mean (SD), y	NA	1.5 (1.0)	NA	NA	1.2 (1.3)	NA
Alcohol-related variables						
Age at first drink, mean (SD), y	16.6 (2.9)	15.3 (3.7)	.43	16.6 (3.0)	15.0 (4.4)	.45
Age when regular drinking began, median (IQR), y	18.0 (18.0-22.0)	20.5 (18.5-21.0)	.56	18.0 (18.0-22.0)	20.0 (18.0-21.0)	.65
Drinking d/mo in last 6 mo, median (IQR), d	2.2 (2.0-3.3)	1.0 (0.7-2.2)	.15	3.3 (2.0-4.3)	1.5 (0.8-2.2)	.13
No. of drinks per drinking d in last 6 mo, mean (SD)	2.4 (1.1)	1.5 (0.9)	.08	2.6 (1.1)	1.9 (0.8)	.21
Alcohol pharmacokinetics						
Peak BAC, mean (SD), g × L^−1^	0.7 (0.1)	0.1 (0.2)	.002	0.8 (0.1)	0.9 (0.1)	.02
Tmax, median (IQR), min^c^	25.2 (18.0-40.2)	21.0 (15.0-24.0)	.05	25.2 (15.0-34.8)	21.0 (21.0-25.2)	.74
Area under the BAC time curve, mean (SD), g × L^−1^min_(0-210)_	95.7 (15.2)	121.8 (21.0)	.005	94.3 (14.2)	126.7 (20.4)	.005
Alcohol elimination rate, mean (SD), g hr^−1^	8.9 (2.1)	8.3 (1.5)	.48	9.4 (2.1)	7.9 (1.7)	.16

Abbreviations: BAC, blood alcohol concentration; BMI, body mass index (calculated as weight in kilograms divided by height in meters squared); NA, not applicable; Tmax, time-to-peak.

^a^ Subsets of participants matched on Tmax to control for gastric emptying time.

^b^ Data on alcohol pharmacokinetics from a subsample of these SG participants were included in Acevedo et al,^2^ 2020.

^c^ From the time of the first sip of alcoholic beverage, consumed over 10 minutes.

The study was conducted in a private room in CFH or UIUC. After fasting overnight, participants completed an oral challenge (0.5 g of alcohol per kg of fat-free mass) and an alcohol clamp session approximately 1 week apart. For the oral challenge, an IV catheter was inserted into a hand vein to obtain serial arterialized venous blood samples, the alcohol dose was ingested over 10 minutes, and BAC was measured using headspace-gas chromatography as previously described.^2^ For the clamp, an IV catheter was inserted into the antecubital vein to infuse 6%v/v alcohol in half-normal saline. Using the computer-assisted alcohol infusion system,^6^ a target breath alcohol concentration of 60 mg/dL was achieved at 15 minutes and maintained for 135 minutes. AER was estimated from the steady state portion of the last 40 minutes of the clamp.^6^ We used analyses of variance and Kruskal-Wallis test to compare pharmacokinetic parameters and other outcomes between groups (eMethods in the Supplement).

## Results

Compared with the control group, the SG group had a shorter Tmax, higher peak BAC, and greater area under the curve (AUC) but a similar AER (Figure, A; Table). In the subset of participants who were matched for Tmax to control for gastric emptying rate, the AUC was increased by 34% (95% CI, 17%-52%) in the SG group (Figure, B; Table).

**Figure.**
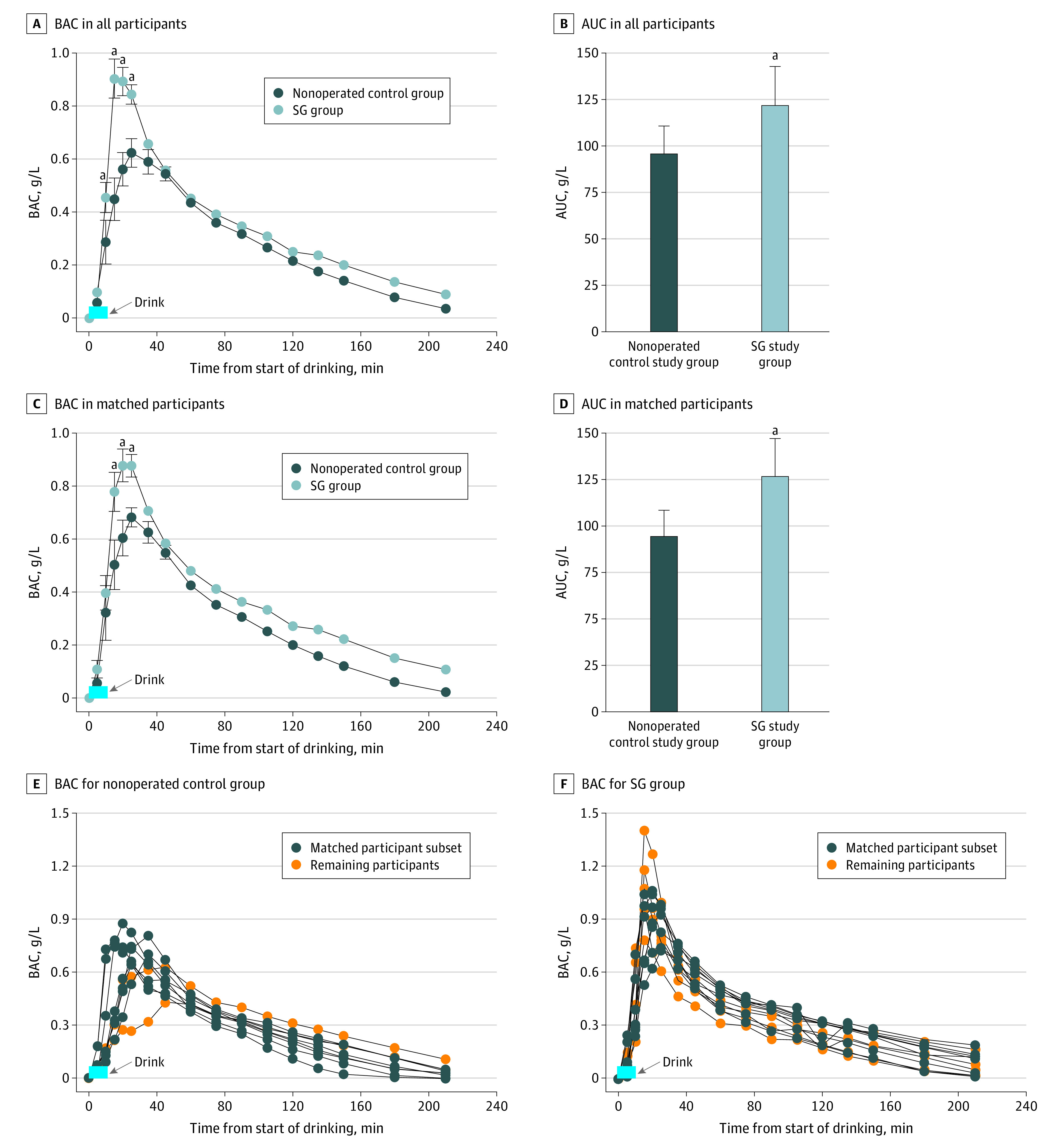
Blood Alcohol Concentrations (BACs) for Sleeve Gastrectomy (SG) Group and Nonoperated Control Group A, SG group included 12 women, and control group included 9 women. B and D, Area under the curve (AUC) for concentration-time curves for both groups. Whiskers indicate SEM. C, SG and control group participants were matched on time-to-peak BAC. Analysis was conducted among 7 women from each group. E and F, Individual BAC curves. ^a^Value significantly different from control group at *P* < .05.

## Discussion

The findings of this study suggest that alcohol FPM occurs in the stomach in women. Despite the overnight fast, which minimizes alcohol FPM, the bioavailability of ingested alcohol increased by 34% in women who had undergone SG compared with women who had not undergone surgery. The increased bioavailability was not explained by systemic AER or rate of gastric emptying; differences between groups remained when matching Tmax. These data help clarify where alcohol FPM occurs and provide a plausible mechanism for the observed increased in alcohol-related disease^3,4^ after bariatric surgery. Given that most patients who undergo metabolic surgery are women, this evaluation included solely women, which is a limitation of the study. Studies in male patients who have undergone SG are needed to better understand sex differences in FPM.

## Notes

Supplement. **eMethods.** Additional Information on Participants, Oral Alcohol Challenge, and Statistical Analysis **eFigure.** Flowchart of Study Participants
eReferences.
