# Error in Table

**DOI:** 10.1001/jamanetworkopen.2022.12773

**Published:** 2022-04-22

In the Research Letter titled “Site of Alcohol First-Pass Metabolism Among Women,”^1^ published March 22, 2022, there was an error in the Table. The peak alcohol concentration in the group with sleeve gastrectomy should have read 1.0 g × L^−1^. This article has been corrected.^1^
