# Supplementary material for: Site of Alcohol First-Pass Metabolism Among Women
Source: JAMA Netw Open. 2022 Mar 22;5(3):e223711. doi: 10.1001/jamanetworkopen.2022.3711 (PMC8941354; doi:10.1001/jamanetworkopen.2022.3711)
Supplement: Supplement. — eMethods. Additional Information on Participants, Oral Alcohol Challenge, and Statistical Analysis eFigure. Flowchart of Study Participants eReferences. [file jamanetwopen-e223711-s001.pdf]

Supplemental Online Content

Seyedsadjadi N, Acevedo MB, Alfaro R, et al. Site of alcohol first-pass metabolism among women. *JAMA Netw Open.* 2022;5(3):e223711. doi:10.1001/jamanetworkopen.2022.3711

eMethods. Additional Information on Participants, Oral Alcohol Challenge, and Statistical Analysis

eFigure. Flowchart of Study Participants

eReferences.

This supplemental material has been provided by the authors to give readers additional information about their work.

© 2022 Seyedsadjadi N et al. *JAMA Network Open.*

eMethods. Additional Information on Participants, Oral Alcohol Challenge, and Statistical Analysis

Participants. Thirty-eight women signed the consent form to participate in the study. Twenty-four participants were women who underwent sleeve gastrectomy and 14 were candidates for the non-operated control group. We included only women because 81% of the patients undergoing bariatric surgery are women[1]. Of these, 11 were excluded because were not meeting inclusion criteria and 1 was a loss to follow up. Data from five participants in the sleeve gastrectomy group were lost because participants dropped out after one study visit (n=2) or due to technical problems with IV access or the computer alcohol infusing system (n=3). Accordingly, 9 participants in the non-operated control group and 12 in the sleeve gastrectomy group completed the study (Supplemental eFigure Study flow chart). All participants completed a screening evaluation consisting of medical history, urine pregnancy test, standard blood test and the assessment of alcohol and other drugs use by interviewing participants with the alcohol and family history assessment modules of the Semi-structured Assessment of the Genetics of Alcoholism[2]. Eligibility criteria was used to determine eligibility for participation. All participants provided written informed consent before participating in the study. The study was approved by the University of Illinois at Urbana-Champaign and Carle Foundational Hospital Institutional Review Boards.

Recruitment. For participants who were evaluated 1-5 years post sleeve gastrectomy, a Carle Foundation Hospital data engineer conducted a filtered search of Carle's Electronic Medical Record applying the inclusion criteria and send out an IRB-approved recruitment letter to potential participants with instructions to contact the PI and her team for more information. Participants in the non-operated control group were recruited from the community using online announcements "eWeek" and social media advertising the research study. The eWeek is a weekly email newsletter with brief summaries of campus announcements of general interest to the members of the University of Illinois at Urbana-Champaign.

Eligibility Criteria. Inclusion criteria for women in the surgery group: 1) aged 21 to 64 years; 2) drink at least 1 standard drink per month but no more than 7 standard drinks per week; 3) underwent sleeve gastrectomy 1-5 years ago; and 4) provided written informed consent. For women in the non-operated control group: 1) did not undergo any bariatric surgery; 2) age, race and body mass index equivalent to women in the surgery group and 3) provided written informed consent.

Exclusion criteria for both groups: 1) having anemia or having donated blood less than 8 weeks before enrolling in the study; 2) being pregnant or breastfeeding; 3) smoking or having quit smoking less than two months ago; 4) having currently an alcohol use disorder or regular use of drugs of abuse; 5) evidence of significant organ system dysfunction (e.g., kidney disease, liver disease, cancer less than five years ago); or 6) taking any medication that might affect alcohol metabolism.

Matching procedures. Women in the Non-Operated Control group were prospectively recruited to be equivalent in BMI, age and alcohol drinking patterns. Potential participants were first phone screened and only invited to enroll in the study if their age, BMI, and alcohol drinking patterns fell within the ranges and distribution of the Sleeve Gastrectomy group in those same outcome variables. To achieve two subgroups with a similar median tmax, we excluded from the sample two women in the Non-Operated Control group whose tmax occurred at 45 min from start of drinking and five women in the sleeve gastrectomy group whose tmax occurred at 15 min from start of drinking (**eFigure**).

© 2022 Seyedsadjadi N et al. *JAMA Network Open.*

This study has been reported in accordance with the Strengthening the Reporting of Observational Studies in Epidemiology (STROBE) reporting guideline.

Body composition. Body fat mass and fat-free mass were determined by using dual-energy X-ray absorptiometry.

Alcohol oral challenge session. Participants arrived at the Carle Hospital or Clinical Suite in Bevier Hall at the UIUC at ~ 8:00 am having been instructed to abstain from alcohol for 36 hours and food for 12 hours. After rechecking non-pregnancy status with a urine pregnancy test, an IV line attached to a three-stopcock, to allow for blood collection, was inserted into a superficial dorsal hand vein. After IV insertion, the hand was heated in a hotbox (50°C) to obtain arterialized venous blood[3] and the catheter was kept patent by slow infusion of saline. Participants were seated throughout the entire collection period. At approximately 9:30 am, participants consumed a standard dose of alcohol (0.5 grams per kg of fat free mass). The alcoholic drink was prepared as a 20% vol/vol solution of 190 proof ethanol mixed with an unsweetened fruity flavored juice (Kool-Aid, Kraft Heinz Company, Chicago, IL) sweetened with Splenda (Heartland Consumer Products, Carmel IN). The drink was provided in two equally divided aliquots and participants consumed each aliquot within consecutive 5-minute periods (i.e., 10 min total). Blood samples were taken at 0, 5,10, 15,20, 25, 35,45, 60, 75, 90, 105, 120, 135, 150, 180 and 210 min from the start of first sip of drinking. Blood alcohol concentrations (BAC) were measured by gas chromatography as previously reported[4] and as briefly described below.

Alcohol clamp session. Participants arrived at the Carle Hospital or Clinical Suite in Bevier Hall at the UIUC at ~ 8:00 am having been instructed to abstain from alcohol for 36 hours and food for 12 hours. After rechecking non-pregnancy status with a urine pregnancy test, an indwelling IV catheter was inserted into the antecubital vein of an arm to infuse 6% v/v alcohol prepared in 0.5% normal saline. Using the Computer-assisted Alcohol Infusion System (CAIS), a target breath alcohol concentration of 60 mg/dL was achieved at 15 min and maintained for 135 min. CAIS calculates the infusion rate based on each participant's estimated total body water (derived from height, weight, age, and gender). Serial breath alcohol concentrations (BrAC) were measured using a breath analyzer "Alcotest 5510 (Drager; Irving, TX) every ~ 5 min to ensure that the BrAC are within 5mg% of the target and to enable minor adjustments to the infusion rates to overcome errors in parameter estimation and experimental variability[5]. At the end of the 135 min, the infusion was terminated, and participants were provided with a meal. BrAC were obtained at several times and participants were not allowed to leave the research facility until their BrAC registered zero.

Gas chromatographic analysis to determine BAC. In brief, blood samples obtained during the alcohol oral challenge session were fractionated to obtained two 100 µl samples. These samples were placed in micro vials containing 50 µl of butanol (51 mg/100ml), used as internal standard. The preparation of the samples was performed in containers filled with crushed ice. Micro vials were hermetically sealed in preparation for headspace GC. The volatile component of the samples was injected by an automatic headspace sampler (G1888A; Agilent 7697A) into the gas chromatograph (Hewlett-Packard 6890; Agilent 7890B)[4].

Classical pharmacokinetic measures. From the raw BAC data, we determined time-to-peak BAC, peak BAC, and area under the BAC time curve (AUC; g/L/min$_{0\text{-}210}$). AUCs were estimated using the trapezoidal rule. Alcohol elimination rates (AER) were estimated from the steady-state

© 2022 Seyedsadjadi N et al. *JAMA Network Open.*

portion of the last 40 min of the alcohol clamp.[5] The alcohol iv clamp provides an estimate of AER that is independent of variations in alcohol absorption processes[5].

Statistical analyses. The primary dependent variables were BAC achieved across time after the consumption of alcohol and classical pharmacokinetic parameters (time-to-peak BAC (tmax), Peak BAC, AUC, AER). We also analyzed subject characteristics (age, body weight, body mass index, fat free mass, age of 1st drink, age at which they started to drink regularly, number of drinking days per month within the last 6 months, number of drinks consumed in a typical drinking day within the last 6 months). Before analyzing the data, we evaluated the distributional properties of the outcome measures for normality, using Shapiro-Wilk and Kolmogorov-Smirnov test and equal variance, using the Levine's Test of Equality. To determine the differences between groups (Sleeve gastrectomy group and Non-Operated Control group) on BAC achieved across time after alcohol consumption, a mixed ANOVA was conducted, with group as the between factor and time since alcohol consumption as a within-subject factor. The rest of the outcome variables were compared with one-way ANOVA, with exception of age at which they started to drink regularly, number of drinking days per month and tmax that were not normally distributed and were therefore compared with Kruskal Wallis H test. A P value ≤.05 was considered statistically significant. For the Mixed ANOVA, the significant interaction for group x time was further evaluated with a post-hoc Fisher's Least Significant Difference. All analysis were performed with SPSS version 27 for windows and STATISTICA version 13.5 (Tibco Software Inc., Palo Alto, CA, USA).

Calculation of %difference in alcohol bioavailability between groups. We calculated the percent differences in the AUC between the groups that were tmax matched and calculated the 95% confidence interval for the two independent samples by using the pooled estimated of the common standard deviation as shown in the formula below:

$$(\bar{x}_1 - \bar{x}_2) \pm t\ S_p \sqrt{\frac{1}{n_1} + \frac{1}{n_2}}$$

Using the t-table, one tailed, with 12 degrees of freedom (i.e., n_1+n_2-2)

© 2022 Seyedsadjadi N et al. *JAMA Network Open.*

eFigure. Flowchart of Study Participants

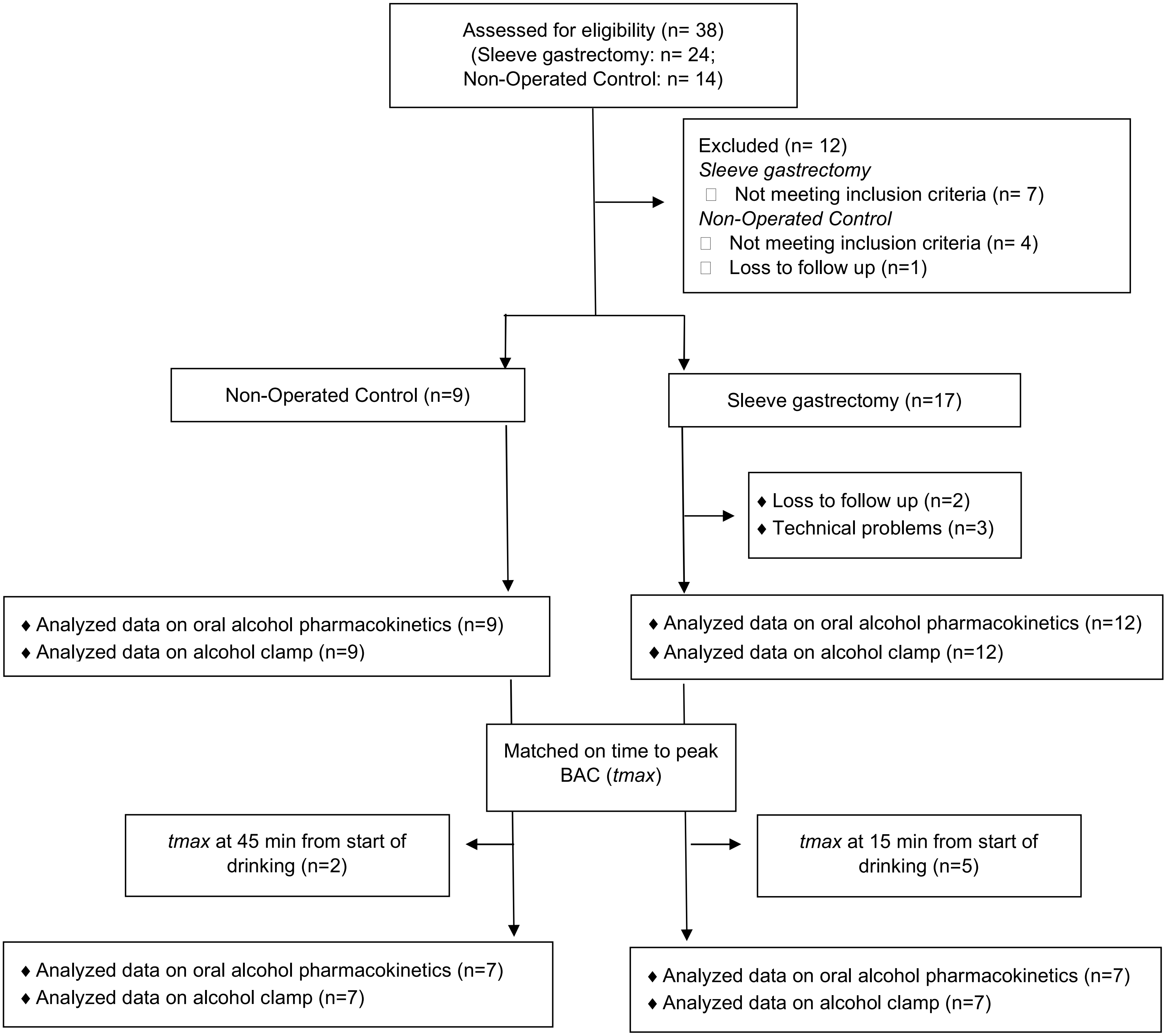

The study population consisted of women who had undergone a sleeve gastrectomy procedure (SG group) within the last 5 yrs. at Carle Foundation Hospital in Urbana, IL and an age and body mass index equivalent control group of women. All recruited women were regular light drinkers (drink at least one standard drink per month but ≤seven standard drinks per week and <four standard drinks per drinking occasion) and had no evidence of binge drinking one month before enrolling in the study. We based our sample size calculation using data reported by Klockhoff et al. 2002[6]. The study was conducted between June 2017 and September 2021.

© 2022 Seyedsadjadi N et al. *JAMA Network Open.*

eReferences.

1. Martin M, Beekley A, Kjorstad R, et al. Socioeconomic disparities in eligibility and access to bariatric surgery: a national population-based analysis. Surg Obes Relat Dis 2010;6:8-15.
2. Bucholz KK, Cadoret R, Cloninger CR, et al. A new, semi-structured psychiatric interview for use in genetic linkage studies: a report on the reliability of the SSAGA. Journal of studies on alcohol. 1994;55:149-58.
3. Sonnenberg GE, Keller U. Sampling of arterialized heated-hand venous blood as a noninvasive technique for the study of ketone body kinetics in man. Metabolism 1982;31:1-5.
4. Acevedo MB, Eagon JC, Bartholow BD, Klein S, Bucholz KK, Pepino MY. Sleeve gastrectomy surgery: when 2 alcoholic drinks are converted to 4. *Surg Obes Relat Dis*. 2018;14(3):277-283.
5. Ramchandani VA, O'Connor S. Studying alcohol elimination using the alcohol clamp method. Alcohol Res Health. 2006;29(4):286-290.
6. Klockhoff H, Naslund I, Jones AW. Faster absorption of ethanol and higher peak concentration in women after gastric bypass surgery. Br J Clin Pharmacol 2002;54:587-91.

© 2022 Seyedsadjadi N et al. *JAMA Network Open.*
